# Ang (1–7) Protects Islet Endothelial Cells from Palmitate-Induced Apoptosis by AKT, eNOS, p38 MAPK, and JNK Pathways

**DOI:** 10.1155/2014/391476

**Published:** 2014-04-02

**Authors:** Li Yuan, Chun-Li Lu, Ying Wang, Yang Li, Xiao-Ya Li

**Affiliations:** Department of Endocrinology, Union Hospital, Tongji Medical College, Huazhong University of Science and Technology, Wuhan 430022, China

## Abstract

This study aimed to explore the effect of angiotensin (1–7) (Ang (1–7)) on palmitate-induced apoptosis in islet endothelial cells and the mechanism of action. MS-1 cells were treated with palmitate in the presence or absence of Ang (1–7). The percentage of apoptotic cells was determined by DNA fragmentation and flow cytometry. Reactive oxygen species (ROS) production was measured using a Reactive Oxygen Species Assay Kit. Expression of AKT, eNOS, C-Jun N-terminal kinase (JNK), and p38 was detected by western blotting. Compared with palmitate treated group, palmitate-induced apoptosis was decreased in MS-1 cells which were preincubated with Ang (1–7) (*P* < 0.05). Palmitate decreased the phosphorylation of AKT and eNOS, and Ang (1–7) increased the phosphorylation of these kinases (*P* < 0.05), with a concomitant reduction in MS-1 cells apoptosis. Ang (1–7) also inhibited the palmitate-induced ROS production and attenuated the apoptosis-related signaling molecule JNK and p38 activation (all *P* < 0.05). PI3K/AKT, eNOS, p38 MAPK, and JNK inhibitors blocked the antilipoapoptosis of Ang (1–7) (all *P* < 0.05). Our findings suggest that Ang (1–7) reduces palmitate-induced islet endothelial cells apoptosis. AKT/eNOS/NO signaling and JNK and p38 pathway are involved in the Ang (1–7)-mediated modulation of islet endothelial cells lipoapoptosis.

## 1. Introduction

Pancreatic islets have a dense capillary network. Intraislet capillaries are lined by fenestrated endothelial cells [1]. Each *β*-cell is surrounded by at least one islet endothelial cell, which may provide signals for islet cell development [2] and important for adult *β*-cell proliferation [3]. Endothelial dysfunction was observed in Orientals with insulin resistance and prediabetic population [4], which is a potential contributor to the pathogenesis of diabetes mellitus. Approaches that improve endothelial function, such as treatment with statins, angiotensin-converting enzyme inhibitors (ACEI), angiotensin receptor blockers (ARB), or peroxisome proliferator-activated receptor gamma ligands (PPAR-*γ*), have been shown to prevent diabetes disease [5]. Maintaining survival and function of islet endothelial cells is a major goal of diabetes risk reduction.

Recently, a homologue of angiotensin converting enzyme (ACE), namely, ACE2 has been identified [6]. ACE2 cleaves angiotensin II (Ang II) to the heptapeptide fragment Ang (1–7), which opposes many of the actions of Ang II [7, 8]. ACE2 is highly expressed in vascular endothelial cells [7, 9]. Previous studies demonstrated that endothelial cells are capable of generating Ang (1–7) from its precursors Ang I and Ang II [10]. The receptor of Ang (1–7), Mas, is also constitutively expressed in human endothelial cells, which provides a physiological basis for the effects of Ang (1–7) in endothelial cells [11]. Many studies have demonstrated that upon activation of ACE2/Ang (1–7)/Mas axis could improve endothelial function [11, 12].

Lo et al. [13] reported that Renin-angiotensin system (RAS) blockade normalized renal ACE2 expression and urinary Ang (1–7) levels and prevented renal proximal tubular cell apoptosis in type 1 diabetic Akita angiotensinogen-transgenic mice. Studies of alveolar epithelial cells confirmed the effect of Ang (1–7) to prevent the Ang II- or endoplasmic reticulum stress-induced apoptosis [14, 15]. More recent work showed that ACE2/Ang (1–7)/Mas axis was involved in mediating apoptosis of different types of cells, but very few direct measurements have been published regarding the antiapoptotic mechanism of ACE2/Ang (1–7)/Mas axis.

In this context, the aim of the present study was to evaluate the hypothesis that Ang (1–7) attenuates palmitate-induced apoptosis in islet endothelial cell. Furthermore, the mechanisms of antilipoapoptosis action of Ang (1–7) were investigated.

## 2. Materials and Methods

### 2.1. Cell Culture

Palmitate, BSA, and KRBH were purchased from Sigma. The islet microvascular endothelial cell line (MS-1) cells, which were purchased from Chinese Academy of Sciences Cell Bank, were cultured in basal medium consisting of Dulbecco's modified Eagle's medium (DMEM, Hyklong, USA) with 10% fetal bovine serum (gibco, USA), 100 U/mL penicillin, and 100 *μ*g/mL streptomycin under standard conditions (5% CO_2_ and 37°C). Cells were synchronized in FBS-reduced media (0.5%) for 12 h prior to experiments then exposed to 0.25% (wt/vol) BSA (CON group) or 0.25 mmol/L palmitate in BSA for 24 h (PA group). Ang (1–7) (PA + Ang (1–7) group) or Mas receptor antagonist A-779 (PA + Ang (1–7) + A779 group) was applied 30 min before palmitate treatment.

### 2.2. DNA Fragmentation

DNA fragmentation activity in MS-1 cells was quantified using a Cell Death Detection kit ELISA plus (Roche Diagnostics, Germany) in accordance with the manufacturer's instructions. Briefly, cells were treated with palmitate and Ang (1–7) as indicated. Following treatment, the cells were washed twice with PBS and incubated with lysis buffer for 20 min at room temperature. After centrifugation to remove nuclei and cellular debris, the supernatants were diluted 1 : 5 with lysis buffer, and each sample was analyzed by ELISA.

### 2.3. Detection of Apoptosis by Flow Cytometry

After the indicated treatment with palmitate and Ang (1–7), cells were harvested and the rate of apoptosis was determined by flow cytometry with an Annexin V-FITC/PI assay (BD Pharmingen, Franklin Lakes, NJ) according to the manufacturer's instructions.

### 2.4. Nitric Oxide Measurement

MS-1 cells were stimulated with palmitate (0.25 mmol/L) in the presence of Ang (1–7) (10^−6^ mol/L) or not. At the end of the incubation, supernatants were collected and NO was determined according to the manufacturer's instructions (Nanjing Jiancheng Bioengineering Institute, China). In some experiments, cells were preexposed to the eNOS inhibitor L-NAME. All the experiments were performed in triplicate.

### 2.5. Real-Time PCR

Total RNA of cultured MS-1 cells was extracted using the TRIzol reagent (Takara, Japan) according to the manufacturer's instructions. 500 ng total RNA was used for cDNA synthesis using cDNA reverse transcription kit (Takara, Japan). The real-time PCR, which was contained in a final volume of 10 *μ*L, consisted of 1 *μ*L cDNA and components of SYBR-Green Supermix (Takara, Japan) according to the manufacturer's instructions. The PCR was carried out in 96-well plates using the ABI Prism 7900HT Sequence Detection System. For ACE2, the primer sequences were 5′-CCGTTGTTTGACTGGCTGAAAG-3′ (forward) and 5′-GCAACAGATGATCGGAACAGG-3′ (reverse); for Mas, the prime sequences were 5′-GCTTGAGGCTATCTTCCTGTGATC-3′ (forward) and 5′-GAGGCTTCCAAACTCAGTCAGTC-3′ (reverse); for GAPDH, the prime sequences were 5′-GGTGAAGGTCGGTGTGAACG-3′ (forward) and 5′-CTCGCTCCTGGAAGATGGTG-3′ (reverse).

### 2.6. Western Blot

Cells were harvested and lysed in a cell lysis buffer (Beyotime Institute of Biotechnology, China) and an additional protease inhibitor cocktail tablet at 1 : 100 final buffer volumes. Protein samples (30 *μ*g) were separated by 8% SDS-polyacrylamide gel electrophoresis and transferred onto pure nitrocellulose membranes (0.45 mm, Bio-Rad Laboratories). Membranes were blocked and incubated with one of the specific antibodies overnight at 4°C. Blots were probed with peroxidase-conjugated goat anti-mouse IgG (Pierce, USA) or peroxidase-conjugated goat anti-rabbit IgG (Amersham, UK) for 1 h at room temperature followed by chemiluminescence detection (Amersham). Antibodies against phosphorylated Ser473-AKT, total AKT, and phosphorylated Ser1177-eNOS were purchased from Cell Signaling Technology; ACE and ACE2 were purchased from Abcam; total eNOS, phosphorylated Thr183/Tyr185 JNK, total JNK, phosphorylated Thr180/Tyr182 p38 MAPK, total p38, AT1R, Mas receptor, and *β*-actin were purchased from Santa Cruz Biotechnology.

### 2.7. Intracellular Reactive Oxygen Species Measurement

The quantitation of intracellular ROS was measured using the fluorescent probe 2,7-dichlorofluorescein diacetate (DCFH-DA), as previously described [16]. Briefly, the MS-1 cells were pretreated with Ang (1–7) (10^−6^ mol/L) for 30 min followed by exposure to palmitate (0.25 mmol/L) for 24 h. After exposure, the cells were washed twice with phosphate buffered saline (PBS) and incubated with culture medium containing 20 *μ*M DCFH-DA (Beyotime Institute of Biotechnology, China) for 20 min at 37°C in the dark. Subsequently, the cells were lysed with lysis buffer, and carboxy-DCF fluorescence in cell lysates was measured by a multimode microplate reader (Bio-Rad, USA), with excitation at 488 nm and emission at 530 nm. The fluorescence intensity was normalized against the protein concentration of each individual well.

### 2.8. Statistical Analysis

Each experiment was repeated at least three times. The data were expressed as mean ± SEM. Statistical analysis was performed with GraphPad Prism 5.0 (San Diego, CA, USA). Statistical differences between groups were analyzed by ANOVA. *P* < 0.05 was considered a statistically significant difference.

## 3. Results

### 3.1. Effect of Palmitate on ACE2 and Mas Expression in MS-1 Cells

**Figure 1:**
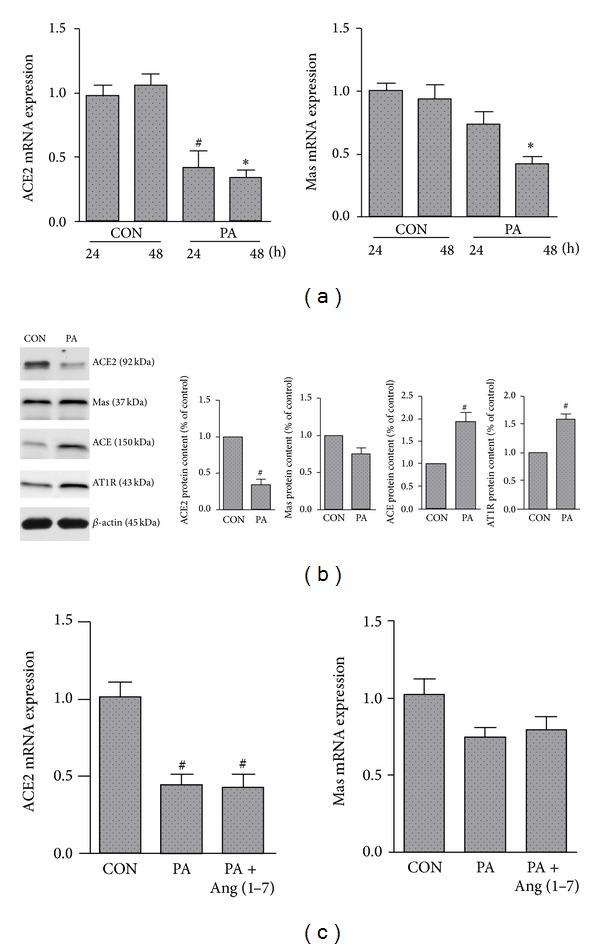
The effects of palmitate on the RAS in cultured MS-1 cells. Cells were incubated for 24 or 48 h with 0.25% BSA (CON) or palmitate (PA, 0.25 mmol/L). ACE2 and Mas receptor mRNA levels were measured (a). Cells were incubated for 24 h with palmitate (PA, 0.25 mmol/L), alone or in combination with Ang (1–7) (10^−6^ mol/L). Representative western blots showing the protein expression levels of ACE2, Mas, ACE, and AT1R (b). RT-PCR showing the mRNA levels of ACE2 and Mas receptor (c). Values are mean ± SEM, *n* = 4 for each group. ^#^ *P* < 0.05 versus CON of 24 h; *P < 0.05 versus CON of 48 h.

As shown in Figure 1(a), real-time PCR results showed that ACE2 mRNA level decreased significantly after exposure to palmitate for 24 or 48 h compared to control cells. In addition, the Mas receptor mRNA level in palmitate treated cells also decreased significantly. Western blot results showed that ACE2 protein level decreased significantly after exposing the cell to palmitate for 24 h (*P* < 0.05). Mas protein level also decreased; however, the difference was not statistically significant. By contrast, ACE and AT1 protein level was increased significantly after palmitate exposure (Figure 1(b)) (both *P* < 0.05). As shown in Figure 1(c), Ang (1–7) did not significantly affect the ACE2 and Mas receptor mRNA level in palmitate treated cells, although the Mas receptor mRNA level was slightly greater in PA + Ang (1–7) group than in PA group.

### 3.2. Ang (1–7) Decreased MS-1 Cells Lipoapoptosis

**Figure 2:**
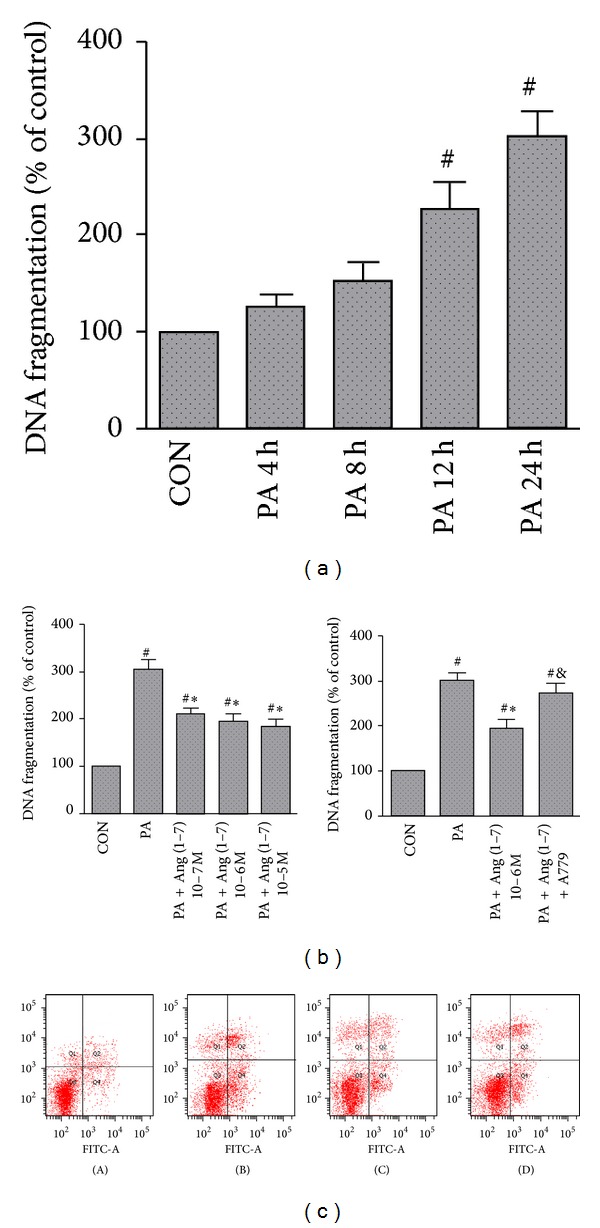
Ang (1–7) blocked palmitate-induced apoptosis in MS-1 cells. Lipoapoptosis depends on the exposure time (a). DNA fragmentation (b) and flow cytometry (c) measured in MS-1 cells exposed to palmitate (0.25 mmol/L) for 24 h, alone or in combination with Ang (1–7) (10^−6^ mol/L) or A-779 (10^−5^ mol/L). (c)(A): CON group; (c)(B): PA group; (c)(C): PA + Ang (1–7) group; (c)(D): PA + Ang (1–7) + A779 group. Values are mean ± SEM, *n* = 4 for each group. ^#^ *P* < 0.05 versus CON; *P < 0.05 versus PA; ^&^ *P* < 0.05 versus PA + Ang (1–7).

Compared with the control group, palmitate significantly enhanced apoptosis in MS-1 cells in a time-dependent manner, with a maximal effect achieved at 24 h (Figure 2(a)). By contrast, palmitate-exposed cells coincubated with Ang (1–7) at various concentrations (10^−5^–10^−7^ mmol/L) decreased DNA fragmentation compared with cells in palmitate group (Figure 2(b)). Incubating the cells with palmitate and Ang (1–7) in the presence of Mas receptor antagonist A779 led to loss of Ang (1–7) protection against lipoapoptosis (Figure 2(b)).

Compared with control cells, treatment with palmitate for 24 h increased the apoptosis of MS-1 cells as evidenced by results of Annexin V-FITC/PI assays (*P* < 0.05). Pretreatment of cells with Ang (1–7) reduced the rate of apoptosis compared with the group treated with palmitate alone (*P* < 0.05). The antilipoapoptosis effect of Ang (1–7) was blocked by A779 (Figure 2(c)).

### 3.3. The Antiapoptotic Action of Ang (1–7) Was Mediated by Activation of AKT-Dependent Signaling Pathways

**Figure 3:**
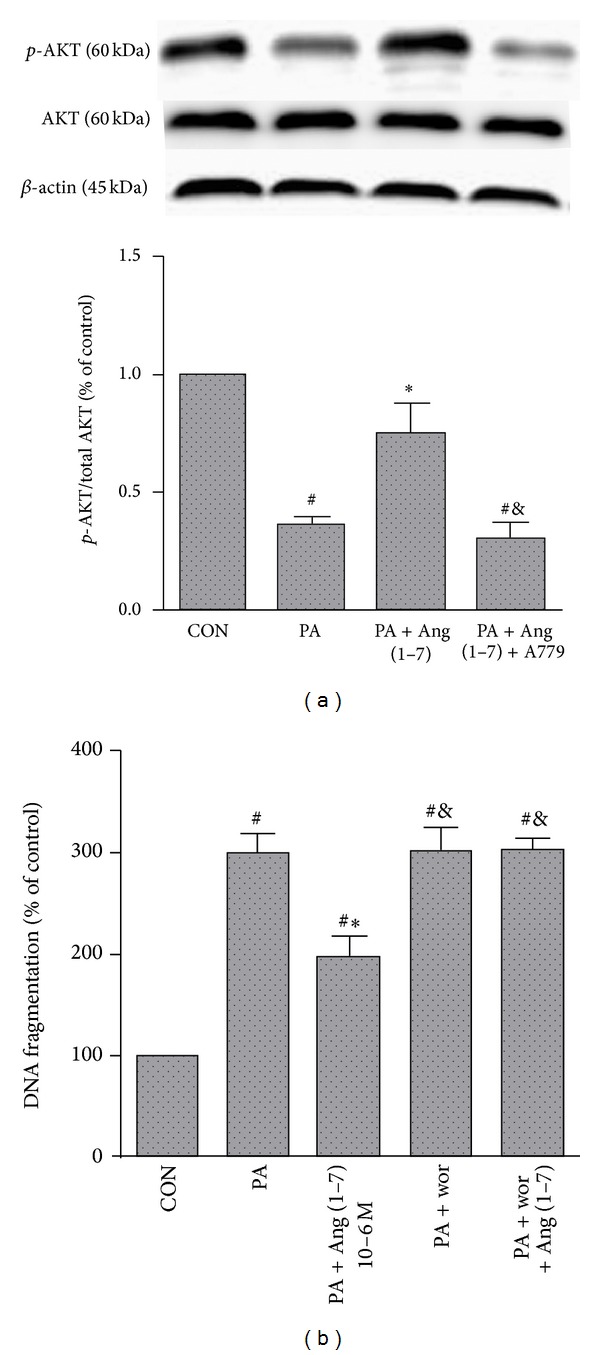
Effect of AKT pathway on antilipoapoptosis effect of Ang (1–7). AKT phosphorylation was measured in MS-1 cells which were exposed to palmitate (0.25 mmol/L) for 24 h, alone or in combination with Ang (1–7) (10^−6^ mol/L) and A779 (10^−5^ mol/L) (a). Wortmannin (10^−6^ mol/L) blocked the antilipoapoptosis effect of Ang (1–7) (b). Values are mean ± SEM, *n* = 3 for each group. ^#^ *P* < 0.05 versus CON; *P < 0.05 versus PA; ^&^ *P* < 0.05 versus PA + Ang (1–7).

To explore the possible mechanism of the antiapoptotic effect of Ang (1–7) on MS-1 cells, specific Ang (1–7)-triggered signaling events, AKT activation was investigated next. As shown in Figure 3(a), palmitate decreased the phosphorylation of AKT (Ser473) by 35.9% as compared with the control group (*P* < 0.05). Pretreatment of cells with Ang (1–7) promoted the phosphorylation of AKT (Ser473) by 74.6% as compared with the control group, higher than that observed in PA group (*P* < 0.05). The protective effect of Ang (1–7) was inhibited by A779 (*P* < 0.05). We next examined the role of AKT in the prevention of apoptosis. As seen in Figure 3(b), Wortmannin countered the protection against lipoapoptosis induced by Ang (1–7) (*P* < 0.05), indicating that this antiapoptotic effect of Ang (1–7) involves AKT signaling pathways.

### 3.4. The Protective Effect of Ang (1–7) Against Lipoapoptosis Was Mediated by eNOS Activation

**Figure 4:**
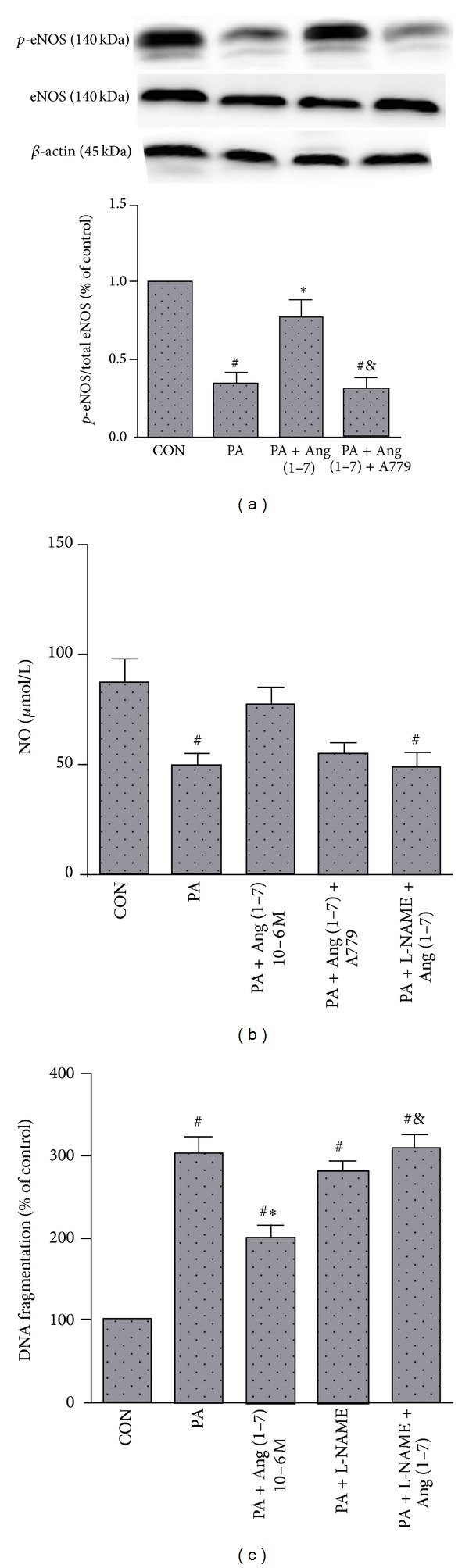
Effect of eNOS/NO pathway on antilipoapoptosis effect of Ang (1–7). eNOS phosphorylation (a) and NO production (b) were measured in MS-1 cells which were exposed to palmitate (0.25 mmol/L) for 24 h, alone or in combination with Ang (1–7) (10^−6^ mol/L) and A779 (10^−5^ mol/L). L-NAME (10^−7^ mol/L) blocked the antilipoapoptosis effect of Ang (1–7) (c). Values are mean ± SEM, *n* = 3 for each group. ^#^ *P* < 0.05 versus CON; *P < 0.05 versus PA; ^&^ *P* < 0.05 versus PA + Ang (1–7).

The PI3K/AKT signaling pathway is known to regulate eNOS activity, and activation of AKT has been shown to stimulate phosphorylation of eNOS at Ser1177 [11]. As shown in Figure 4(a), palmitate decreased the phosphorylation of eNOS by 33.6% as compared with the control group (*P* < 0.05). Ang (1–7) prevented this negative effect by increasing the phosphorylation of eNOS by 76.1% (*P* < 0.05). To confirm the involvement of eNOS in modulation of Ang (1–7)-dependent antiapoptotic effect, studies were carried out in the presence of the specific eNOS inhibitor L-NAME. L-NAME had no noticeable effects on cellular apoptosis but prevented the ability of Ang (1–7) to suppress apoptosis induced by palmitate (*P* < 0.05) (Figure 4(c)).

### 3.5. Ang (1–7) Increased NO Production under Palmitate Condition

To determine whether Ang (1–7)-induced activation of AKT/eNOS could affect downstream NO release, the production of NO in media was investigated. As can be seen in Figure 4(b), incubation of MS-1 cells with palmitate reduced the NO production by 43.5%, as compared with the control group (*P* < 0.05), and this inhibition was partly reversed when cells were pretreated with Ang (1–7). Mas receptor antagonist A-779 blocked the effect of Ang (1–7). We next incubated cells with L-NAME, an inhibitor of NOS. The protective effect of Ang (1–7) on NO production was completely abolished by L-NAME. These results indicated that the protective effect of Ang (1–7) enhance NO production in MS-1 cells through receptor Mas, which is mediated by eNOS activation.

### 3.6. Ang (1–7) Decreased Palmitate-Induced ROS Production in MS-1 Cells

**Figure 5:**
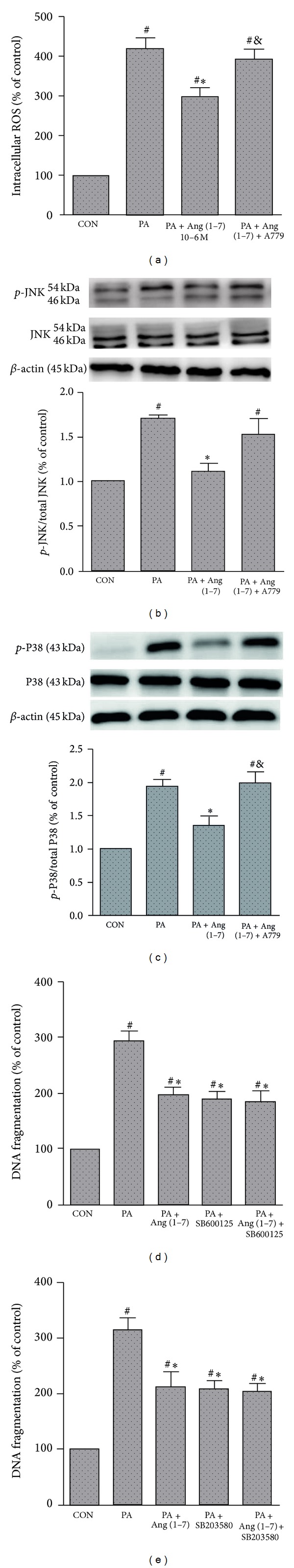
Effect of JNK and P38 pathway on antilipoapoptosis effect of Ang (1–7). Cells were incubated in palmitate (0.25 mmol/L) for 24 h with or without Ang (1–7) (10^−6^ mol/L), A-779 (10^−5^ mol/L), the JNK inhibitor SP600125 (2 × 10^−5^ M) or the p38 MAPK inhibitor SB203580 (10^−5^ M). ROS production (a), JNK phosphorylation (b), P38 phosphorylation (c), and DNA fragmentation were measured (d, e). Values are mean ± SEM, *n* = 3 for each group. ^#^ *P* < 0.05 versus CON; *P < 0.05 versus PA; ^&^ *P* < 0.05 versus PA + Ang (1–7).

As it is known that excess reactive oxygen species can damage cellular DNA, lipids, and protein, thereby elicit apoptosis. We further decided to examine the influence of palmitate and Ang (1–7) on intracellular ROS production in the MS-1 cells. Exposure of MS-1 cells to palmitate for up to 24 h induced a statistically significant increase in ROS levels (*P* < 0.05). However, Ang (1–7) treatment markedly inhibited the ROS production, and the effect was blocked by the addition of Mas receptor antagonist A-779 (both *P* < 0.05) (Figure 5(a)).

### 3.7. Effect of Ang (1–7) on Palmitate-Induced MAPK Phosphorylation in MS-1 Cells

The JNK Ser/Thr kinase is widely recognized as a key signaling node in the regulation of cell survival in response to stress-activated apoptosis [17]. Other MAPK proteins P38 can be also activated by cytokines or cellular stress and participate in the regulation of cell survival. Further experiments showed that exposure of MS-1 cells to media containing palmitate increased JNK phosphorylation by 1.71-fold as compared with control cells (*P* < 0.05). Application of Ang (1–7) prevented palmitate-induced increase in JNK phosphorylation (*P* < 0.05) and effect of Ang (1–7) was partly blocked by A779 (Figure 5(b)). The similar change was observed on p38 MAPK. Ang (1–7) blocked palmitate-induced p38 phosphorylation increase (*P* < 0.05). A779 blocked the effect of Ang (1–7) on reversing palmitate-induced increase in p38 phosphorylation (*P* < 0.05) (Figure 5(c)). In contrast, Ang (1–7) did not show obvious effect on palmitate-induced increase in ERK phosphorylation (data not shown).

### 3.8. The Antilipoapoptosis Effect of Ang (1–7) Is Mediated by p38 MAPK- and JNK-Dependent Mechanisms

To clarify whether blocking P38 MAPK and JNK activation affected the protective effect of Ang (1–7) against lipoapoptosis, we pretreated MS-1 cells with SP600125 (2 × 10^−5^ M), a JNK inhibitor, or SB203580 (10^−5^ M), a p38 MAPK inhibitor, for 1 h prior to palmitate and Ang (1–7) treatment. Results showed that palmitate induced a robust increase in apoptosis, which was attenuated by Ang (1–7). Pretreatment with SB600125 (Figure 5(d)) or SP203580 (Figure 5(e)) significantly reduced cellular apoptosis induced by palmitate (both *P* < 0.05). By contrast, when cells were incubated with palmitate and Ang (1–7) in the presence of SP600125 or SB203580, the antilipoapoptosis effect of Ang (1–7) was blocked (both *P* < 0.05).

## 4. Discussion

The ability of ACE2/Ang (1–7)/Mas axis to mediate lipid metabolism has been observed in a number of experimental systems. Treated with Ang (1–7), rats with diabetic cardiomyopathy [18] and diabetic nephropathy [19] have a significant reduction in dyslipidemia in a Mas-dependent way. In addition, transgenic animals with increased plasma levels of Ang (1–7) had decreased triglycerides and cholesterol levels [20]. Contrary, Mas-knockout mice on the FVB/N background have impaired lipid metabolism, leading to dyslipidemia [21]. In the study in cultured islet endothelial cells, our results showed that ACE2/Ang (1–7)/Mas axis was suppressed under hyperlipid condition, which was manifested as downregulation in ACE2 and Mas expressions.

Plasma concentrations of free fatty acids (FFAs) are increased in states including hypertension [22], obesity [23], and diabetes [24]. It is known that FFAs are putative mediators of cellular apoptosis. Our current islet endothelial cell culture studies demonstrated that Ang (1–7) attenuated the proapoptotic effect of palmitate via the activation of the Mas receptor, as evidenced by the reduction in its inhibitory effect in the presence of Mas receptor antagonist A-779, but the molecular mechanisms of this protection remain to be elucidated.

Several studies have reported that eNOS plays a pivotal role in endothelial cell proliferation and survival [25]. Long-term exposure to FFAs has been shown to trigger apoptosis in human endothelial cells and also reduce eNOS protein levels [26]. It is well established that the actions of Ang (1–7) include activation of eNOS, release of NO, and this effect is dependent on the activation of the PI3K/AKT pathway [11]. In our present study, we found that palmitate decreased the phosphorylation of AKT and eNOS. Pretreatment of MS-1 cells with Ang (1–7) prevented the palmitate-induced decrease in these kinases, which may contribute to the survival response mediated by Ang (1–7). The induction of apoptosis in MS-1 cells by the simple addition of Wortmannin or L-NAME provided direct evidence in support of this theory.

NO is released from the endothelium following stimulation of the endothelial NO-synthase. Previous studies have shown that NO either endogenously produced or exogenously applied in physiologically relevant concentrations acts as an endothelial cell survival factor [27, 28]. Clinical studies have demonstrated that the production of NO is impaired in the presence of high circulating levels of FFA [29, 30], in line with the findings of this study showing exposure of MS-1 cells to palmitate substantially reduced NO production. However, pretreatment of MS-1 cells with Ang (1–7) prevented the palmitate-induced decrease in NO production. Together, these findings establish that AKT/eNOS/NO signaling pathway largely contributes to the antilipoapoptosis effect of Ang (1–7) in MS-1 cells.

A series of researches have shown that FFAs stimulate ROS formation in endothelial cells [16, 31]. ROS, which decrease the antioxidant capacity and/or oxidative DNA repair capacity of the cell, are known to induce apoptotic cell death in various cell types, including endothelial and smooth muscle cells [32]. With diabetic nephropathy mice model, Moon et al. [33] showed that Ang II-induced NAD(P)H oxidase activation and ROS production are negatively modulated by Ang (1–7) in mesangial cells. Our results are consistent with these findings, showing that treatment with Ang (1–7) reduced palmitate-induced ROS production and decreased the amount of apoptotic cells. Therefore, the reduction of ROS may contribute to the ability of Ang (1–7) to attenuate lipoapoptosis of islet endothelial in culture. The earlier demonstration that Ang (1–7) is protective against neuronal apoptosis which was accompanied by reduced brain NAD(P)H oxidase expression in hypertensive diabetic rats [34] lends support to the interpretation.

Palmitate was reported to increase apoptosis of human endothelial progenitor cells via p38 and JNK mitogen-activated protein kinase pathways [35]. In line with those results, the present study found that palmitate increased the phosphorylation of both p38 MAPK and JNK in MS-1 cells and inhibition of p38 MAPK and JNK with specific inhibitors completely abolished the proapoptotic effects of palmitate, suggesting that palmitate induces apoptosis in MS-1 cells through p38 MAPK- and JNK-dependent pathways. It is also consistent with the requirement for JNK phosphorylation in the induction of apoptosis through the mitochondrial pathway [36]. FFAs induce apoptosis through the mitochondrial pathway [37].

Our study revealed that Ang (1–7) significantly decreased the phosphorylation levels of p38 MAPK and JNK. Interestingly, the antiapoptotic effect of Ang (1–7) was also abolished by specific inhibitors of p38 MAPK and JNK, indicating that activation of these kinases not only contributes to increased lipoapoptosis but also that their suppression plays an important role in MS-1 cells survival caused by Ang (1–7). Recent studies in type 2 diabetic mice model support that a critical role of Ang (1–7) attenuating ROS-mediated injury by attenuating MAPK activation [33] provides indirect evidence in support of this conclusion. ROS is known to damage cells indirectly by modifying the activity of p38 MAPK and JNK signaling pathways [38]. Other studies have shown that excessive and/or prolonged ER stress activated the P38 MAPK and JNK pathway [39]. It has been reported that Ang II upregulated ER chaperones and induced apoptosis in cultured neonatal rat cardiomyocyte [40]. The present study has confirmed the significantly increased ACE and AT1 levels in the palmitate treated MS-1 cells. On the other hand, the AT1R specific blocker-olmesartan reported to attenuate ER stress-induced renal apoptosis via the modulation of JNK-Caspase12 pathway [41]. In contrast, Ang (1–7) completely abrogated the ER stress-induced apoptosis of alveolar epithelial cells [15]. These results combined together suggest that the antilipoapoptosis effect of Ang (1–7) involve p38 MAPK and JNK signaling pathways.

In summary, our results suggest that downregulation in ACE2/Ang (1–7)/Mas axis in RAS is involved in palmitate-induced islet endothelial cells apoptosis. Reinforcing the effect of ACE2/Ang (1–7)/Mas axis by application of Ang (1–7) attenuates palmitate-induced islet endothelial cells apoptosis. We further suggest that AKT/eNOS/NO signaling pathway, JNK, and p38 in MAPK pathway involve the antilipoapoptosis effect of Ang (1–7) in islet endothelial cells.
